# Multidisciplinary Approaches to Better Understanding the Health Implications of Migration Across the Life-Course

**DOI:** 10.1093/geroni/igaf122.1865

**Published:** 2025-12-31

**Authors:** Yesenia Cruz-Carrillo, Frank Infurna, Kyriakos Markides

## Abstract

Migration influences health and aging trajectories across the life course. The goal of this symposium is to bring together a collection of papers that utilize multidisciplinary perspectives on trajectories and social determinants of health among middle-aged and older adult immigrants in the United States and Mexico. Cruz-Carrillo and Infurna use data from the Health and Retirement Study (HRS) to examine historical changes in and explanatory factors of self-rated health, episodic memory, and depressive symptoms; successive cohorts of immigrants demonstrated historical improvements compared to earlier-born cohorts. García and Garcia use data from the American Community Survey to investigate geographic variation in cognitive functioning among Latino immigrants across 24 states. The probability of reporting cognitive difficulties was higher among those in Northeastern states compared to other regions; differences by Latino heritage groups were also observed. Sheftel and colleagues use data from the HRS to estimate the projection of poverty and uninsurance rates among unauthorized Latino immigrants. By age 76, 56% of unauthorized foreign-born are expected to be in poverty and 59% uninsured. Saenz and Burns use data from the Mexican Health and Aging Study to examine differences in cognition levels among individuals from rural areas who stayed and those who migrated. The cognitive advantages of urban-dwelling inform our understanding of healthy migration. The discussion by Markides will integrate the four papers by highlighting novel findings that contribute to our understanding of migration across the life course and insights gained on the implications of population-level health trends in the U.S. and Mexico.

